# Novel E3 Ubiquitin Ligases That Regulate Histone Protein Levels in the Budding Yeast *Saccharomyces cerevisiae*

**DOI:** 10.1371/journal.pone.0036295

**Published:** 2012-05-03

**Authors:** Rakesh Kumar Singh, Melanie Gonzalez, Marie-Helene Miquel Kabbaj, Akash Gunjan

**Affiliations:** Department of Biomedical Sciences, College of Medicine, Florida State University, Tallahassee, Florida, United States of America; Texas A&M University, United States of America

## Abstract

Core histone proteins are essential for packaging the genomic DNA into chromatin in all eukaryotes. Since multiple genes encode these histone proteins, there is potential for generating more histones than what is required for chromatin assembly. The positively charged histones have a very high affinity for negatively charged molecules such as DNA, and any excess of histone proteins results in deleterious effects on genomic stability and cell viability. Hence, histone levels are known to be tightly regulated via transcriptional, posttranscriptional and posttranslational mechanisms. We have previously elucidated the posttranslational regulation of histone protein levels by the ubiquitin-proteasome pathway involving the E2 ubiquitin conjugating enzymes Ubc4/5 and the HECT (*H*omologous to *E*6-AP *C-T*erminus) domain containing E3 ligase Tom1 in the budding yeast. Here we report the identification of four additional E3 ligases containing the RING (*R*eally *I*nteresting *N*ew *G*ene) finger domains that are involved in the ubiquitylation and subsequent degradation of excess histones in yeast. These E3 ligases are Pep5, Snt2 as well as two previously uncharacterized Open Reading Frames (ORFs) YKR017C and YDR266C that we have named Hel1 and Hel2 (for *H*istone *E*3 *L*igases) respectively. Mutants lacking these E3 ligases are sensitive to histone overexpression as they fail to degrade excess histones and accumulate high levels of endogenous histones on histone chaperones. Co-immunoprecipitation assays showed that these E3 ligases interact with the major E2 enzyme Ubc4 that is involved in the degradation related ubiquitylation of histones. Using mutagenesis we further demonstrate that the RING domains of Hel1, Hel2 and Snt2 are required for histone regulation. Lastly, mutants corresponding to Hel1, Hel2 and Pep5 are sensitive to replication inhibitors. Overall, our results highlight the importance of posttranslational histone regulatory mechanisms that employ multiple E3 ubiquitin ligases to ensure excess histone degradation and thus contribute to the maintenance of genomic stability.

## Introduction

Histones are essential basic proteins that package the genomic DNA of all eukaryotes into nucleosomes to form chromatin [1], [2], [3]. In doing so, histones also regulate access to the genetic information contained within the DNA and thereby affect all aspects of DNA metabolism. Most histones are encoded by multiple genes in all eukaryotes [4] that have the potential for generating histones in excess of the requirement for chromatin assembly [5]. Any excess of the positively charged histones can allow them to potentially associate non-specifically with negatively charged molecules such as DNA in the cell, resulting in deleterious effects on cell viability and genomic stability [6], [7], [8]. As such, in organisms such as the *Xenopus* that undergo rapid embryonic cell cycles in the absence of transcription, large quantities of histones are stored bound to chaperone proteins such as Nucleoplasmin in their oocytes to provide adequate quantities of histones for chromatin assembly during DNA synthesis [9], [10]. A related strategy has also been reported in *Drosophila* and other fly embryos wherein histones appear to be stored in lipid droplets [11]. In the budding yeast and mammalian cells, histone chaperones such as Asf1 (Anti-Silencing Function 1), CAF-1 (Chromatin Assembly Factor-1), Spt16 (Suppressor of *Ty* 16) and Nap1 (Nucleosome Assembly Protein 1) can bind to and buffer against only a limited excess of histone proteins [6], [12], [13], [14], [15], [16]. Therefore, to avoid the problems associated with excess histone accumulation, most eukaryotic cells rely largely on the strict regulation of their histone protein levels via transcriptional, posttranscriptional, translational and posttranslational mechanisms [5], [17].

Proteins are the structural and functional workhorses of all cells. In order to respond effectively to constantly changing environmental cues, protein levels must be regulated by eliminating the pools that have either been damaged or are simply no longer required for normal cell function. Therefore, cells have evolved regulated proteolysis to fine-tune their metabolic processes [18], [19]. This regulated proteolysis involves a fairly well defined choreography known as the ubiquitin-proteasome pathway. Ubiquitin is a short 76-amino acid peptide that is universally conserved among eukaryotes (hence the name ubiquitin). Ubiquitin is attached to substrate proteins to mark them for destruction or transduce other signals in the cell. Several ubiquitin molecules (at least four) need to be covalently attached to the same lysine residue in the target protein for it to be recognized and degraded by the multifunctional protease known as the 26S proteasome [20]. The conjugation of ubiquitin to proteins requires the sequential action of three enzymes. The ubiquitin-activating enzyme (Enzyme 1 or simply E1; Uba1 in the budding yeast) hydrolyzes ATP to convert ubiquitin into an activated form, which is covalently linked at its carboxyl terminus to a cysteine (Cys) residue of the E1 enzyme via a high-energy thiol-ester linkage [21]. The activated ubiquitin molecule is then transferred to the second enzyme of this pathway, an ubiquitin conjugating enzyme (Ubc or E2; Ubc1–Ubc13 in the budding yeast), and the activated form is maintained through the formation of a thiol-ester linkage with a Cys residue in the E2 enzyme. The third enzyme in the process, the ubiquitin ligase (E3; approximately 80 E3 ligases in yeast), supports the transfer of ubiquitin to substrates and generally provides the specificity [20], [22], [23]. E3 ubiquitin ligases have been broadly classified into three main categories, namely RING (*R*eally *I*nteresting *N*ew *G*ene) finger domain containing E3 ligases [24], the U-box domain containing E3 ligases that carry a modified RING domain [25] and HECT (*h*omologous to *E*6-AP *C-t*erminus) domain containing E3 ligases [26]. The RING domain is a Cys rich sequence motif that can bind two zinc atoms and the majority of the E3 ligases belong to this class [24]. The consensus RING motif has been defined as a unique pattern of Cys and histidine (His) residues at defined positions in a peptide sequence that is Cys-X_2_-Cys-X_9–39_-Cys-X_1–3_-His-X_2–3_-Cys-X_2_-Cys-X_4–48_-Cys-X_2_-Cys (and is often abbreviated to Cys_3_-His-Cys_4_), where X can be any amino acid. The RING domain has been shown to facilitate the interaction between the E2 and the substrate being ubiquitylated without ever being covalently attached to ubiquitin. U-box E3 ligases contain a U-box motif which is similar to RING motif except that it lacks the canonical Cys residues for zinc coordination [22], [25]. The consensus 75-amino acid U-box domain is poorly conserved and is characterized by the same sequence of -helices, ß-sheets and unstructured loops found in RING fingers. The HECT E3 ligases form a relatively small family of conserved E3 ligases that are present from yeast to humans [26], [27]. These ligases are characterized by the HECT domain, a C-terminal region of approximately 350 amino acids with significant similarity to the C-terminus of E6-AP. Unlike RING domain and U-box E3 ligases, the HECT E3 ligases interact with the cognate E2 ubiquitin conjugating enzymes leading to covalent attachment of the ubiquitin moiety by a thiol-ester bond to a conserved Cys residue in HECT domain. Subsequently this ubiquitin is transferred directly from the E3 ligase to the substrate protein. Elongation of the initial ubiquitin into polyubiquitin chains is carried out by the concerted actions of E2 and E3 enzymes. Even though some E2s might provide the chain-type specificity, most of the substrate specificity is achieved by the E3 ligases. Not surprisingly, cells have a large number of proteins in their proteome (1–2% of the total number of proteins) that are putative E3 ligases [23]. The importance of E3 ligases is underscored by the fact that a number of human diseases including cancer, neurodegenerative processes, metabolic disorders, familial Parkinson's disease and Angelman's syndrome have been found to have defects in this proteolytic pathway [28], [29], [30], [31]. Further, some of the most important cell cycle regulators are under very tight regulation and this is achieved by employing multiple E3s for this task. The most studied protein to fall in this class is the tumor suppressor p53, which is known to be ubiquitylated by at least 11 different E3 ligases (Mdm2, Pirh2, Cop1, TOPORS, Synoviolin, CHIP, UBC13, CARP1, CARP2, p300/CBP and ARFBP1) [32]. Even though these E3 ligases have other targets, they all contribute individually in regulating p53 levels, which is vital for proper cell cycle progression.

We have been engaged for the past decade in trying to understand the posttranslational regulation of histone protein levels using the budding yeast *Saccharomyces cerevisiae* as a model system. Histones are generally considered to be extremely stable proteins with half-lives in the order of several months in mammalian cells [33]. However, these half-lives are likely to mainly reflect the contribution of chromatin bound histones, as we have previously shown that non-chromatin bound histones are rapidly degraded with a half-life of 30–40 minutes in yeast cells [6], [7]. We went on to demonstrate that the phosphorylation, ubiquitylation and subsequent degradation of excess histones in yeast occurs via the ubiquitin-proteasome pathway. We identified the DNA damage checkpoint kinase Rad53 as the master regulator of this pathway which also employs the E2 ubiquitin conjugating enzyme Ubc4/5 and the HECT domain E3 ligase Tom1 for the degradation related polyubiquitylation of excess histones. Interestingly HUWE1 (HECT, UBA and WWE domain containing 1), the human homolog of Tom1, has been reported to ubiquitylate all four core histones *in vitro* [34] as well as control the levels or activities of important cellular regulators such as Cdc6 [35], Mcl-1 [36], Myc [37] and p53 [38]. *tom*1 deletion strains are defective in efficient degradation of ectopically expressed histone H3 [7]. Subsequent experiments carried out by us strongly suggested that additional E3 ligases maybe involved in the degradation of excess histones in budding yeast. Here we report the identification and characterization of four additional histone E3 ligases, namely Pep5, Snt2 and two novel E3 ligases that correspond to previously uncharacterized ORFs. These ORFs (YKR017c on chromosome XI and YDR266c on chromosome IV) encode RING finger domain containing proteins. Based on their involvement in ubiquitylating histones, we propose the name *HEL1* and *HEL2* respectively (for *H*istone *E*3 *L*igase) for these two ORFs. Using a combination of genetic and biochemical assays we show that these genes encode genuine ubiquitin E3 ligases and histones serve as at least one of their major substrates. As such, these E3 ligases play an important role in regulating histone protein levels and are likely to contribute to the maintenance of genomic stability in the budding yeast.

## Results

### Identification of four RING finger containing predicted E3 ligases that are sensitive to histone overexpression

**Figure 1:**
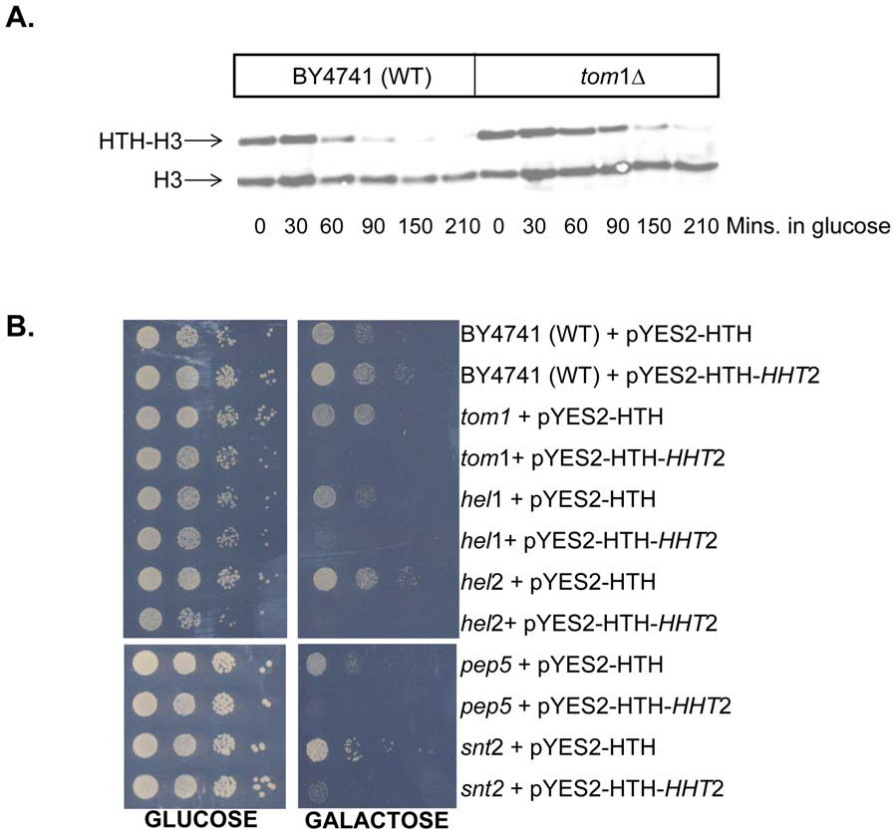
Identification of strains lacking specific E3 ligases that confer sensitivity to histone overexpression. (**A**) *The tom1 deletion strain can degrade exogenous histones with much slower kinetics than the wild type strain*. Histone degradation assay was carried out exactly as described previously [7] whereby a galactose inducible, HIS10-TEV-HA (HTH) tagged histone H3 was expressed in G1 arrested cells for 90 minutes, following which cells were switched to glucose media and harvested at the indicated time points. Whole cell extracts were prepared and resolved on 18% polyacrylamide gels and subjected to Western blotting with the H3-C antibody described previously [6] that recognizes both the endogenous chromatin associated histone H3 as well as the overexpressed H3. The entire experiment was carried out in G1 arrested cells to prevent incorporation of exogenous histones into chromatin that occurs in replicating cells. (**B.**) *Strains lacking Hel1, Hel2, Pep5 and Snt2 E3 ligases are sensitive to histone overexpression*. The histone overexpression sensitivity screen was carried out exactly as described previously [7]. Briefly, wild type (WT) BY4741 and deletion strains corresponding to the indicated E3 ligases were transformed with either an empty vector (pYES2-HTH) or a galactose inducible HTH tagged histone H3 expressing plasmid (pYES2-HTT-*HHT2*). Then 10-fold serial dilutions of the resulting transformants were plated on glucose (no histone overexpression) or galactose (allows histone overexpression) containing media without uracil to select for the plasmid. Plates were photographed after 3 days of incubation at 30°C.

Although we had previously demonstrated that *tom1* mutants were defective in the degradation of exogenous histones [7], when followed for longer time points, the *tom1* mutants appeared to degrade the exogenously expressed histones, albeit with much slower kinetics compared to the wild type cells (Figure 1A). Additionally, the *tom1* mutant was not as sensitive to genotoxic agents as the *ubc4 ubc5* double deletion strain that lacks both the E2 enzymes responsible for the degradation related ubiquitylation of histones in yeast [7]. These data strongly suggested that additional E3 ligases may be involved in the degradation of excess histones in budding yeast. The yeast proteome has approximately 80 E3 ligases [23] and we used a simple screen based on the sensitivity of yeast mutants defective in the histone degradation pathway to histone overexpression as described previously [7] to identify additional histone E3 ligases. For this, we acquired all the non-essential deletions strains corresponding to the predicted E3 ligases from the yeast genome deletion collection (Open Biosystems). Additionally, we acquired temperature sensitive mutants for a few of the essential E3 ligases as well. All the mutant strains along with the isogenic wild type strain were transformed with either the empty vector (pYES2-HTH) or a galactose inducible HIS10-TEV-HA tagged histone H3 overexpression construct (pYES2-HTH-HHT2) described previously [7]. The transformants were grown overnight on a minimal liquid media with raffinose as the sole source of carbon, but without uracil to maintain selection for the plasmid. Then 10-fold serial dilutions corresponding to each strain were plated on glucose or galactose containing plates lacking uracil and incubated at 30°C (semi-permissive temperature for the temperature sensitive mutants) for 2–3 days prior to being photographed. The growth of each strain was compared between galactose (in the presence of histone overexpression) and glucose plates (no histone overexpression). In addition to the *tom1* deletion strain that served as a positive control in our screen, four putative E3 deletion strains were sensitive to histone overexpression to varying degrees (Figure 1B). Two of the strains corresponded to deletions of previously characterized genes *PEP5* and *SNT2*, while the remaining two strains carried deletions of previously uncharacterized ORFs and we named them *hel1* and *hel2* for *H*istone *E*3 *L*igase. Since histone overexpression causes cytotoxicity via multiple mechanisms in yeast cells [8] we needed to further characterize the four genes identified in our initial screen to determine if they encoded bona fide histone E3 ligases.

### *hel1*, *hel2*, *pep5* and *snt2* mutants are defective in histone degradation

**Figure 2:**
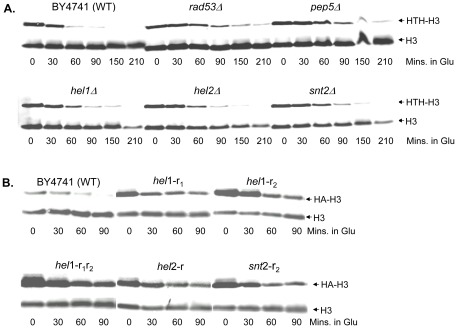
Strains lacking putative E3 ligases Hel1, Hel2, Pep5 and Snt2 are defective in degrading excess histones. (**A**) *Strains carrying deletions of hel1, hel2, pep5 and snt2 are deficient in degrading exogenously expressed histones.* The exogenous histone degradation assay was carried out in the indicated strains carrying the pYES2-HTH-*HHT2* plasmid as described in Figure 1A. The *rad53* deletion strain serves as a positive control in this assay as we have previously shown that it is defective in the degradation of exogenously expressed histones [6]. The endogenous chromatin bound histone H3 is not degraded in this assay and serves as a loading control. Glu = glucose. (**B**) *The RING domains of Hel1, Hel2 and Snt2 are required for efficient degradation of excess histones*. Strains carrying mutations in the RING (*r*) domains of Hel1, Hel2 and Snt2 were generated as described in the Materials and Methods section. These mutant strains were then transformed with the pYES6/CT-HA-*HHT2* plasmid carrying a Blasticidin resistance marker and a galactose inducible, HA-tagged histone H3 gene (HA-H3). The excess histone degradation assay was carried out as described in (A) except that the duration of the experiment was limited to 90 minutes and the exogenous HA-H3 was detected using HA.11 antibodies, while the endogenous H3 was detected using the H3-C antibody described previously [6], [7].

If *hel1*, *hel2*, *pep5* and *snt2* mutants were sensitive to histone overexpression due to a defect in histone degradation, then this defect could be detected using our histone degradation assays described previously [6], [7]. For this assay, G1 arrested cells were induced with galactose for 90 minutes to express HA tagged histone H3, after which cells were switched to glucose media to stop the synthesis of the tagged H3. Samples were harvested at 30-minute or indicated intervals and processed for Western blotting using the H3-C antibody as described previously [6], [7], except that the signals were detected using fluorescently labeled secondary antibodies on a Li-COR Odyssey imager. Compared to the wild type BY4741 strain, the *hel1*, *hel2*, *pep5* and *snt2* deletion strains were clearly defective in degrading ectopically expressed histone H3 to varying degrees, with *pep5* being the most defective and nearly indistinguishable from *rad53* mutants (Figure 2A). The levels of endogenous chromatin bound histone H3 do not change during the course of the experiment and serve as an excellent internal loading control. Next, we investigated if the RING domains of the putative histone E3 ligases were required for the degradation of excess histones. For this we used site-directed mutagenesis to replace the critical Cys and His residues in the RING domains of Hel1, Hel2 and Snt2 with Alanine (Ala) residues as described in Materials and Methods. These *hel*1-r_1_, *hel*1-r_2_, *hel*1-r_1_r_2_, *hel*2-r and *snt2*-r_2_ mutants carrying point mutations in their respective RING domains were as defective in excess histone degradation as the *hel1*, *hel2* and *snt2* deletion strains (Figure 2B), strongly suggesting that their RING domains are important for histone protein regulation.

### *hel1*, *hel2*, *pep5* and *snt2* mutants accumulate excess histones on the histone chaperone Asf1

**Figure 3:**
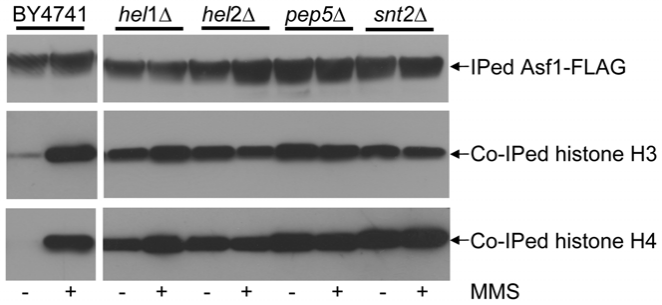
*hel1*, *hel2*, *pep5* and *snt2* deletion strains accumulate excessive amounts of endogenous histones on the histone chaperone Asf1. The chromosomal copy of the gene encoding Asf1 was tagged with a 3×FLAG epitope at the C-terminus in the indicated strains. Whole cell extracts were prepared from 0.5 L cell cultures treated with or without DNA damaging agent Methylmethane Sulfonate (MMS; 0.033%) as described previously [7]. Then, Asf1-FLAG was immunoprecipitated (IPed) using FLAG-M2 antibodies and the co-immunoprecipitated (Co-IPed) histones were analyzed by Western blotting using the H3-C and H4 polyclonal antibodies described previously [6].

We have previously shown that yeast strains defective in the histone degradation pathway such as *rad*53, *ubc*4, *ubc*5 and *tom*1 accumulate excess endogenous histones on histone chaperones such as Asf1 which typically binds small amounts of histones under normal conditions but becomes saturated with histones upon replication arrest or DNA damage [6], [7]. If *hel1*, *hel2*, *pep5* and *snt2* are indeed defective in degrading endogenous histones, they should also exhibit histone chaperones overloaded with endogenous histones under normal conditions. Wild-type and E3-mutant strains carrying FLAG3 tagged Asf1 (Asf1-FLAG) were treated with or without methyl methane sulfonate (MMS) to induce alkylation damage, following which Asf1-FLAG was immunoprecipitated using FLAG-M2 affinity resin. As expected, in the absence of MMS, small amounts of H3 were associated with Asf1–FLAG in wild-type cells, which increased dramatically upon MMS treatment (Figure 3). In contrast, *hel1*, *hel2*, *pep5* and *snt2* deletion strains showed high levels of H3 associated with Asf1–FLAG both in the absence and presence of MMS-induced DNA damage. The near saturation of Asf1 with histones in each of these mutants even in the absence of DNA damage strongly suggests that the corresponding E3 ligases contribute independently of each other in the posttranslational regulation of endogenous histone protein levels.

### Hel1, Hel2, Pep5 and Snt2 interact with Ubc4 and histones

**Figure 4:**
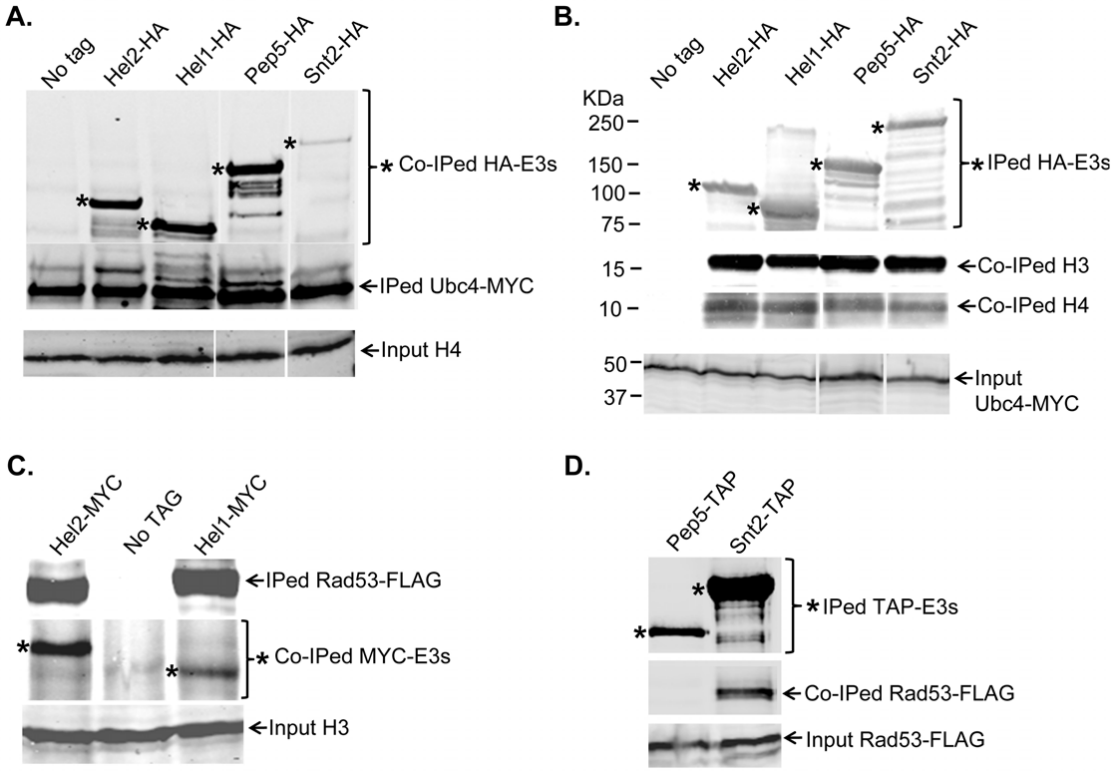
Hel1, Hel2, Pep5 and Snt2 interact with the E2 enzyme Ubc4 as well as histones. (**A**) *Hel1, Hel2, Pep5 and Snt2 interact with Ubc4*. Whole cell extracts prepared from 0.5 liter cultures of the indicated strains were used to IP Ubc4-MYC essentially as described for Asf1-FLAG IPs in Figure 3, but using anti-MYC EZ view beads (Sigma). Co-IPed HA-tagged E3 enzymes were detected using HA.11 antibodies. Apart from the predominant bands corresponding to the full length proteins (indicated by the asterisks), additional bands of weaker intensity are detected and these are likely to be either degradation products (faster migrating) or ubiquitylated (slower migrating) forms of these proteins. No co-IPed proteins were detected in the parental untagged strain carrying Ubc4-MYC but lacking HA-tagged proteins. 1% of the whole cell extracts were loaded on a separate gel to determine that roughly equal amounts of proteins were present in the input fraction by measuring the amount of histone H4. (**B**) *Hel1, Hel2, Pep5 and Snt2 interact with histones*. The HA-tagged E3 enzymes were IPed from 1 liter cultures of the indicated strains using HA.11 antibodies. Co-IPed endogenous histones H3 and H4 was detected using the polyclonal antibodies described previously [6]. No co-IPed histones were detected in the “no tag” control. The amount of Ubc4-MYC was measured in 1% of the input fraction to demonstrate that roughly equal amounts of cell extracts were used for the IP reactions. (**C**) *Hel1 and Hel2 interact with Rad53*. MYC tagged Hel1 or Hel2 were IPed using MYC antibody beads from 3 liter cultures of strains carrying FLAG-tagged Rad53. IPed and Co-IPed proteins were detected by Western Blotting using appropriate antibodies. The relative amount of histone H3 present in each of the “input” extracts used for the IP is shown to demonstrate that same amount of material was used for each IP. (**D**) *Snt2 interacts with Rad53 whereas Pep5 does not*. Tandem Affinity Purification (TAP) tagged Pep5 or Snt2 was IPed from 3 liter cultures of strains carrying FLAG-tagged Rad53. Any co-IPed Rad53 was detected using FLAG antibodies. The similar levels of Rad53-FLAG present in the extracts confirm that the same amount of extracts was used for each IP. No evidence for any interaction between Rad53 and Pep5 was observed even upon scaling up the Pep5-TAP IP by 3-fold (data not shown).

All the new histone E3 ligases identified in our screen are RING finger domain containing proteins. RING domains are known to directly interact with Ubc (E2) enzymes as well as the substrate, thereby helping the Ubc enzymes transfer the ubiquitin to the substrate proteins [24], [39]. Hence, if the putative RING finger histone E3 ligases identified by us are bona fide E3 ligases, they should interact physically with their substrate histones as well as the E2 enzymes involved in the degradation related ubiquitylation of histones. We tested this idea directly by checking if Hel1, Hel2, Pep5 and Snt2 interact with the major histone ubiquitin conjugating enzyme Ubc4 and histones. For this we transformed a strain expressing MYC13-tagged Ubc4 [7] with constructs expressing HIS6-HA-Protein A (HIS6-HA-PrA) tagged E3 ligases. Then we prepared whole cell extracts and immunoprecipitated (IPed) either Ubc4-MYC using anti-MYC EZ View beads (Sigma) (Figure 4A) or HA-tagged E3 ligases using magnetic beads conjugated to HA.11 antibodies (Figure 4B). All IPed material was resolved on precast gradient gels. Western blotting with MYC and HA antibodies clearly shows that all the four E3 ligases co-immunoprecipitated (co-IPed) with Ubc4-MYC (Figure 4A). Similar results were obtained using reciprocal immunoprecipitation experiments (data not shown). Further, both histones H3 and H4 co-IPed with all the four E3 ligases (Figure 4B). Overall, these interactions demonstrate that the new RING finger containing histone E3 ligases can potentially mediate the ubiquitin transfer from the E2 enzyme Ubc4 to the substrate histones by acting as a scaffold to bring these proteins together.

### Hel1, Hel2, Pep5 and Snt2 promote ubiquitylation of histones *in vitro*

**Figure 5:**
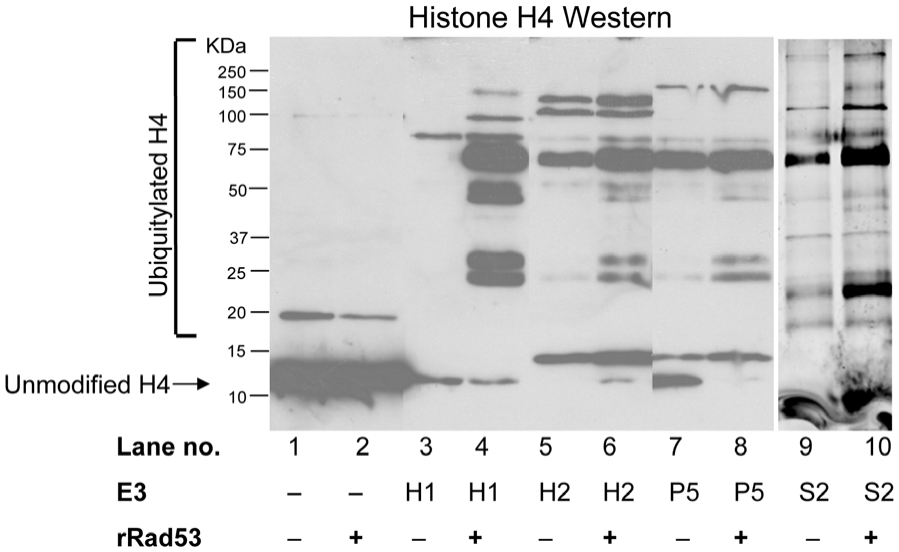
Hel1, Hel2, Pep5 and Snt2 can efficiently ubiquitylate histone H4 *in vitro* in a reaction that is stimulated by Rad53. Recombinant and purified components were used to reconstitute the ubiquitylation of histone H4 *in vitro* exactly as described previously [7]. The reaction products were resolved on an 18% polyacrylamide gel and processed for Western blotting with H4 antibodies. Addition of commercially available recombinant yeast Uba1 (E1), human UbcH5A (E2; homolog of yeast Ubc4) and HIS6-tagged ubiquitin to recombinant human histone H4 with or without Rad53 did not result in appreciable histone modifications, apart from some monoubiquitylation (lanes 2 and 1 respectively). Only the addition of the E3 ligases Hel1 (H1), Hel2 (H2), Pep5 (P5) and Snt2 (S2) purified from yeast extracts via TAP epitope tags resulted in considerable high molecular weight histone modifications, which were further stimulated to varying degrees by the addition of recombinant Rad53 to the reaction mixture.

To further demonstrate that the E3 ligases identified by us were bona fide histone E3 ligases, next we tested if they can polyubiquitylate histones *in vitro*. For this we set up *in vitro histone* ubiquitylation reactions using recombinant E1, E2, ubiquitin and histone H4 along with purified TAP-tagged E3 ligases as described previously [7]. Reactions were stopped by the addition of Sodium Dodecyl Sulfate (SDS) loading buffer and boiling prior to analysis by Western blotting using histone H4 antibodies. The formation of high-molecular weight species clearly demonstrates that histone H4 is efficiently ubiquitylated *in vitro* using Hel1, Hel2, Pep5 and Snt2 E3 ligases (Figure 5). Further, the inclusion of Rad53 significantly stimulated the activity of all these E3 ligases in the *in vitro* ubiquitylation reactions, especially in the case of Hel1 (Figure 5, compare lanes 3 to 4, 5 to 6 and 7 to 8), suggesting that the newly identified E3 ligases may recognize the same Rad53-dependent phosphorylation as degradation signals on histones as the Tom1 ligase identified previously [7].

### Hel1, Hel2, Pep5 and Snt2 do not regulate the levels of the E2 enzyme Ubc4

**Figure 6:**
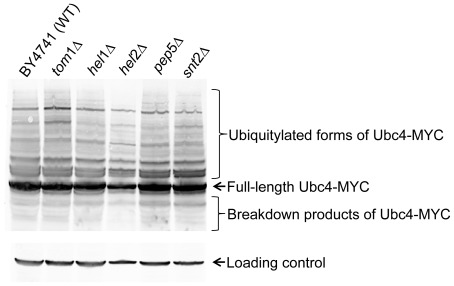
The E3 ligases Hel1, Hel2, Pep5, Snt2 and Tom1 do not regulate the protein levels of Ubc4, the major E2 enyme involved in excess histone degradation. Whole cell lysates were prepared as described previously [69] from wild type and the indicated E3 ligase deletion strains carrying MYC-tagged Ubc4. The lysates were resolved on a 12% polyacrylamide gel and processed for Western blotting using MYC antibodies. No significant differences are observed in the levels of full-length Ubc4-MYC, or in its slower migrating modified forms and faster migrating degradation products.

Although the *in vivo* and *in vitro* data presented here so far strongly suggest that the newly identified E3 ligases are likely to be directly involved in histone ubiquitylation and degradation, it is also formally possible that one or more of these ligases may be indirectly affecting histone levels by controlling the abundance of Ubc4, the major E2 enzyme involved in the ubiquitylation mediated degradation of histones. To investigate this possibility, we measured the levels of full-length MYC13-tagged Ubc4, as well as its ubiquitylated forms and degradation products in wild type and E3 mutant strains. Although we readily detected multiple bands migrating slower than full-length Ubc4-MYC, we did not observe any differences in the levels of the full-length or ubiquitylated forms, as well as in the degradation products of this protein upon the deletion of any of the newly identified E3 ligases (Figure 6). This data strongly suggests that these E3 ligases do not regulate Ubc4 protein levels, although it is still formally possible that these E3 ligases may regulate the levels of Tom1 or each other, thereby indirectly influencing histone levels.

### Histone E3 ligases exhibit varying degrees of sensitivity to genotoxic agents such as the replication inhibitor hydroxyurea

**Figure 7:**
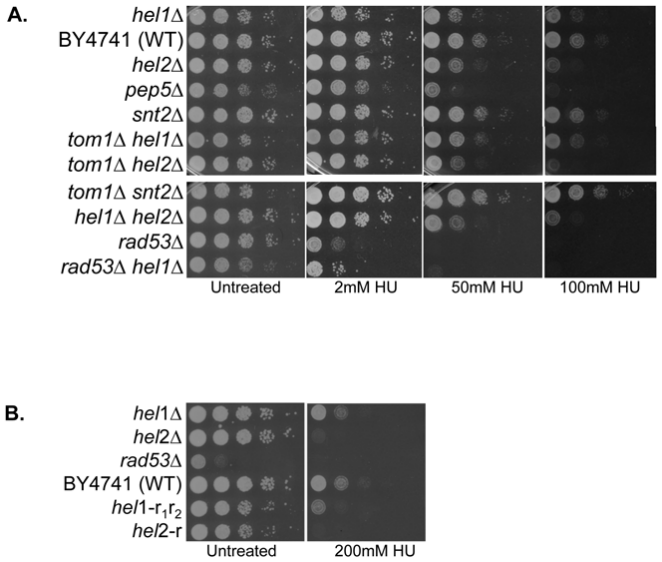
Histone E3 ligase mutants exhibit varying degrees of sensitivity to the replication inhibitor hydroxyurea. (**A**) *hel1, hel2 and pep5 mutants are sensitive to hydroxyurea*. 10-fold serial dilutions of the indicated strains were plated on media containing glucose with or without the indicated concentrations of hydroxyurea (HU) and incubated at 30°C for 3 days before being photographed. (**B**) *Mutations in the RING finger domains of* Hel1 *and* Hel2 *render them sensitive to HU*. 10-fold serial dilutions of the indicated strains were plated on media containing galactose with or without 200 mM HU and incubated at 30°C for 3 days before being photographed. The *hel1-r_1_r_2_* mutant carries inactivating mutations in both *r* finger domains of Hel1, while the *hel2-r* mutant carries inactivating mutations in the single *r* finger domain of Hel2. The *rad53* deletion strain was included as a positive control.

DNA damage in eukaryotes occurs in context of chromatin. Therefore, histone proteins and chromatin structure is likely to influence DNA damage and repair. DNA repair enzymes need to gain access to the damaged DNA in order to repair it. We have previously shown that yeast strains lacking factors involved in the regulation of histone protein levels such as the E3 ligase Tom1 are sensitive to genotoxic agents to varying degrees [7]. To assess if the new histone E3 ligases identified in our screen were also sensitive to genotoxic agents, we plated 10-fold serial dilutions of mutant and wild type strains on plates containing the replication inhibitor hydroxyurea (HU). We found the *pep5* deletion strain to be very sensitive to HU, though not as sensitive as the *rad53* deletion strain, while the *hel1* and *hel2 deletion* mutants were sensitive to HU to a lesser extent (Figure 7A). The *snt2Δ* mutant was not sensitive at all to HU, suggesting the possibility that different histone E3 ligases may be employed for the degradation of excess histones in different contexts. Combining the *tom1* deletion mutant which is slightly sensitive to HU [7] with the *hel1*, *hel2* and *snt2* deletion mutants individually did not further exacerbate the HU sensitivity of any of these E3 ligases (Figure 7A), suggesting that they may be working in the same pathway. Similar results were obtained for the *hel1 hel2* and the *rad53 hel1* double mutants, as the HU sensitivity of the *hel2* and *rad53* single mutants was not enhanced any further in these double mutants (Figure 7A). However, all our attempts to generate the *tom1Δ pep5Δ* double mutant have failed so far, suggesting that this combination may be synthetically lethal (data not shown) and that these two E3 ligases may work in parallel pathways that are redundant with each other. We also investigated if the RING domains of Hel1 and Hel2 played any role in resistance of yeast cells to HU. Mutation of the critical residues within the RING finger domains of Hel1 and Hel2 rendered them as sensitive to HU as the complete loss of the proteins (Figure 7B), once again suggesting that the RING finger domains are essential for the function of these E3 ligases.

## Discussion

In this study we have identified and characterized four putative E3 ligases that contribute to the posttranslational regulation of histone protein levels in the budding yeast. As opposed to the previously identified HECT-domain containing histone E3 ligase Tom1 [7], all the four new histone E3 ligases carry RING finger domains that enable them to polyubiquitylate excess histones for subsequent degradation. Non-degradation related polyubiquitylation of histones H2B has also been reported to occur in the budding yeast [40]. As such, although we have focused here on the involvement of these new E3 ligases in the degradation related ubiquitylation of histones, we acknowledge the possibility that they may also be involved in histone ubiquitylation that is not related to degradation. The five histone E3 ligases identified so far in the posttranslational regulation of histone proteins represent roughly 7% of the 80 predicted E3 ligases in the budding yeast [23]. However, it is possible that this list may expand further upon the analysis of the remaining essential predicted E3 ligases that we have not screened so far. This involvement of multiple E3 ligases in the regulation of histone protein levels is reminiscent of the multiple E3 ligases that independently contribute to the regulation of p53 tumor suppressor protein levels in mammalian cells [32]. Overall, the identification of multiple E3 ligases, each of which appears to contribute independently to the regulation of histone protein levels, highlights the importance of the posttranslational degradation of histones in yeast cells.

Two of the E3 ligases identified in our screen, Hel1 and Hel2, correspond to previously uncharacterized budding yeast ORFs YKR017C and YDR266C respectively. Hel1 is only one of two yeast E3 ligases that belong to the RBR (RING-in between-RING) or TRIAD sub-class of E3 ligases that are characterized by the presence of two RING finger domains with a novel Cys-rich Cys_6_-His-Cys in-between-RING (IBR) domain present in between the two RING domains [41], [42]. The other RBR E3 ligase in the budding yeast, Itt1, is not sensitive to histone overexpression and as such it is unlikely to be involved in the regulation of histone protein levels (data not shown). The RBR family of E3 ligases include human E3 ligases such as the human homolog of *Drosophila* Ariadne (HHARI; also known as ARIH1) that shares homology with Hel1, as well as TRIAD1 (or ARIH2) and Parkin (or PARK2), the latter of which has been implicated in a subset of Parkinson's disease [43]. It remains to be determined if these human RBR E3 ligases that share similarities with Hel1 can ubiquitylate histones. Recent mechanistic insight into these RBR E3 ligases suggest that they may function as hybrid RING/HECT E3 ligases whereby the first RING domain interacts with the E2 enzyme while the second RING domain participates in the transfer of the ubiquitin moiety to the substrate [44]. Not surprisingly, the second RING domain of HHARI appears to have a very different structure compared to canonical RING domains [45]. Mutations in any one of the two RING domains of Hel1 result in similar defects in histone degradation (Figure 2B) and confer the same degree of DNA damage sensitivity as the null mutant (Figure 7B), further confirming that both the RING domains are required for the function of RBR E3 ligases. Overall, our studies on Hel1 constitute the first characterization of an RBR E3 ligase in yeast and suggest that histones are a potential substrate.

Hel2 is a canonical RING domain containing E3 ligase that has been previously shown to be phosphorylated by the DNA damage checkpoint kinase and histone protein regulator Rad53 [46]. Not surprisingly, *in vitro* ubiquitylation of histone H4 in the presence of Hel2 is significantly stimulated by the presence of Rad53 (Figure 5). Further, *hel2* mutants are sensitive to the replication inhibitor HU and this maybe in part due to its role in regulating histone protein levels, although it is quite likely that there are additional substrates that are ubiquitylated *in vivo* by Hel2 in response to replication arrest and we are currently investigating this. The human protein with the highest similarity to Hel2 is an uncharacterized zinc finger protein 598 (ZNF598), although known E3 ligases such as KPC1 (Kip1 Ubiquitination-Promoting Complex 1; also known as RNF123) also show limited similarity to Hel2 [47]. It is unclear if these human E3 enzymes are capable of ubiquitylating histones.

Snt2 is a poorly characterized protein. It was initially identified and named for the presence of the DNA binding SANT (SWI3, ADA2, N-CoR and TFIIIB) domain and has been predicted to function as a transcriptional regulator based largely on *in silico* analyses, although this prediction is yet to be verified experimentally [48], [49]. Snt2 also has three PHD (Plant Homeo-Domain) fingers consisting of the Cys_4_-His-Cys_3_ motif which is typically involved in binding methylated lysine residues on histones, although the Snt2 PHD fingers show weak or nonspecific binding to methylated histone H3 peptides at best [50]. The PHD finger is very similar to the Cys_3_-His-Cys_4_ containing RING finger domain and two of the PHD fingers of Snt2 are also RING fingers potentially capable of E3 ligase activity. Our data clearly show that the *snt2* deletion strain is sensitive to histone overexpression (Figure 1B), defective in histone degradation (Figure 2A) and accumulates excess endogenous histones (Figure 3). Further, point mutations in the second RING domain of Snt2 result in a defect in excess histone degradation (Figure 2B). More importantly, Snt2 interacts with Ubc4, histones and Rad53 (Figure 4) and exhibits robust histone ubiquitylation activity *in vitro* that is further stimulated by Rad53 (Figure 5). Hence, based on these data we conclude that Snt2 is a genuine histone E3 ligase and contributes to the regulation of histone protein levels.

Pep5 (also known as Vps11) was initially isolated as a protease deficient mutant [51]. Pep5 was subsequently shown to be involved in protein trafficking and vacuole biogenesis pathways [52], although it has not been studied as a RING E3 ligase. In our *in vitro* ubiquitylation assay, Pep5 shows robust activity in ubiquitylating histone H4 (Figure 5), indicating that histones may serve as its substrates *in vivo* as well. Of the four histone E3 ligases identified in this study, the *pep5* deletion strain exhibited the most pronounced defects in the degradation of exogenous histones (Figure 2A) and accumulated high amounts of excess endogenous histones on the histone chaperone Asf1 (Figure 3). Interestingly, *pep5* mutants have been reported to exhibit a synthetic growth defect when combined with *hir1*, *hir2* or *asf1* mutations, and are synthetically lethal when combined with *rad53* mutations [53]. Hir1 and Hir2 are part of the four-subunit HIR complex that serves as a negative transcriptional regulator of histone gene expression outside of S-phase in yeast [54], apart from functioning as a histone chaperone [55]. Asf1 also contributes to the transcriptional regulation of yeast histone genes [56], while Rad53 is known to regulate histone protein levels [6], [7]. Taken together, our data suggests that Pep5 is likely to be a major player in the regulation of histone proteins, which would explain the synthetic phenotypes observed upon the simultaneous loss of *pep5* along with known regulators of histone levels. Loss of the histone transcriptional regulator complex HIR may result in an increased production of endogenous histones, thereby increasing the burden on the remaining histone regulators, including Pep5. Similarly, loss of Asf1 would result in a reduction in the capacity of the cells to sequester excess endogenous histones on the remaining histone chaperones, thereby channeling more histones for degradation via Pep5. It will be interesting to determine if Vps11, which appears to be a clear human homolog for Pep5 [57], is also involved in histone regulation.

We have previously provided evidence that the kinase activity of Rad53 is required for the efficient polyubiquitylation of histones both *in vitro* and *in vivo* [7]. We further identified the tyrosine 99 residue of histone H3 as a crucial determinant for the degradation of this histone *in vivo*, presumably due to phosphorylation at this residue. Based on the phosphorylation-ubiquitylation mediated degradation of well-known proteins [58], [59], we proposed a similar model for the degradation of excess histones whereby these histones were first detected and phosphorylated by Rad53, leading to their efficient polyubiquitylation and subsequent degradation by the proteasome. Consistent with this model, our current *in vitro* ubiquitylation reactions demonstrate that the activities of all the identified histone E3 ligases in ubiquitylating histone H4 were further stimulated by the addition of Rad53, but to varying degrees (Figure 5), suggesting that these ligases may also recognize phosphorylation dependent degradation signals on histones similar to Tom1. Further, just like Tom1 [7], Hel1 shows limited H4 ubiquitylation activity in the absence of Rad53, while Hel2, Snt2 and Pep5 show significant H4 ubiquitylation under the same conditions (Figure 5). Upon the addition of Rad53, the H4 ubiquitylation activity of Hel1 was stimulated strongly, while the activities of Hel2, Snt2 and Pep5 showed a more modest increase. The varying degree of Rad53-dependent stimulation of the *in vitro* histone ubiquitylation reactions by these E3 ligases may be indicative of the requirement for additional cellular regulatory factors, such as accessory proteins and possibly other kinases that are missing from our *in vitro* reactions, which may be crucial for the full range of regulated activities of these ligases *in vivo*. Since deletion of *pep5* confers a synthetic lethal phenotype when combined with the *rad53* deletion [53] (and data not shown), it is possible that at least this and possibly all of these E3 ligases may carry out significant histone ubiquitylation independent of Rad53 *in vivo* by relying on other kinases to phosphorylate excess histones and mark them for degradation. This idea would be consistent with the strong defects in histone protein regulation in the *pep5* (Figure 2A and 3) and *rad53* [6], [7] single mutants, the synthetic lethality of the *rad53 pep5* [53] and possibly the *tom1 pep5* double mutants (data not shown), as well as the mutually exclusive localization of Pep5 and Rad53 in the cytoplasm and the nucleus respectively.

Our previous studies have shown that excess histones are degraded by the proteasomes following polyubiquitylation [7]. Since the proteasomes are primarily localized in the nucleus in budding yeast [60], [61], [62], following the initial identification of the nuclear histone E3 ligase Tom1 [7], we had suggested that excess histone degradation was likely to be occurring in the nucleus. However, the cytoplasmic localization of the histone E3 ligases such as Hel2 and Pep5, as well as the nucleocytoplasmic localization of Snt2 [63], suggest the possibility that some excess histone degradation may occur in the cytoplasm (the localization of Hel1 has not been determined yet). Due to the predominant nuclear localization of proteasomes in budding yeast [60], [61], [62], cytoplasmic degradation of excess histones may even occur in a proteasome independent manner, perhaps in the vacuoles. A very recent report suggests that histones may be targeted for degradation via the Chaperone Mediated Autophagy (CMA) pathway in mammalian cells [64]. Although CMA has not been reported to occur in yeast, a related autophagy process known as Vacuolar Import and Degradation (VID) has indeed been described in yeast [65] and this pathway requires components of the ubiquitylation pathway [66], [67]. Hence, it is possible that the known function of Pep5 in vacuolar sorting [52] is somehow involved in such a proteasome independent cytoplasmic degradation of excess histones. Based on their localization, another possibility is that Tom1 targets excess histones in the nucleus for degradation, while Hel2 and Pep5 are involved in the cytoplasmic degradation of excess histones, whereas Snt2 contributes to histone degradation in both cellular compartments. Future studies will determine if this is indeed the case.

## Materials and Methods

All yeast strains used are listed in Table S1. These strains were either obtained from Open Biosystems budding yeast genome deletion collection or were generated in the lab as needed using standard yeast manipulations [68]. MYC13-tagged Ubc4 and the plasmid pRS416-*RAD53*-FLAG for the expression of FLAG-tagged Rad53 have been described previously [7]. TAP-tagged strains were obtained from Open Biosystems. Plasmids pYES2-HTH and pYES2-HTH-*HHT2* have been described previously [7]. Plasmid BG1805 based constructs expressing C-terminal HIS6-HA-Protein A (HIS6-HA-PrA) tagged Hel1, Hel2, Pep5 and Snt2 under the control of a galactose inducible promoter were also obtained from Open Biosystems. Mutagenesis of the *HEL1*, *HEL2* and *SNT2* RING finger domains was carried out on the plasmid BG1805 based constructs expressing Hel1, Hel2 and Snt2 using Stratagene's QuikChange Multi Site-Directed Mutagenesis kit following the manufacturer's instructions. Briefly, the crucial Cys and His residues in the RING (*r*) finger domains were mutated to Ala residues. The *hel1-r_1_* mutant carries the Cys-195, His-197, Cys-200→Ala mutations in *r_1_*, while the *hel1-r_2_* of carries the His-356, Cys-359, Cys-362→Ala mutations in *r_2_*. The *hel1-r_1_r_2_* mutant carries both the *r_1_* and *r_2_* mutations simultaneously. The *hel2-r* mutant carries the Cys-79, His-81, Cys-87→Ala mutations in its *r* finger domain. The *snt2-r_2_* carries the Cys1061, His1066, Cys1069→Ala mutations in its second *r_2_* finger domain. All mutations were confirmed by sequencing the entire mutant gene. The plasmids bearing the *r* finger mutants of the E3 ligases were transformed into cells lacking the endogenous gene for the respective E3 ligase to perform the experiments shown in figures 2B and 7B. All other methods used such as the histone degradation assay, histone overexpression sensitivity assay, Western blotting, co-immunoprecipitation assay and *in vitro* ubiquitylation assay have been described extensively in our previous publications [6], [7].

## Supporting Information

Table S1 **List of strains used in this study.**
(PDF)
