# Supplementary material for: Novel E3 Ubiquitin Ligases That Regulate Histone Protein Levels in the Budding Yeast Saccharomyces cerevisiae
Source: PLoS One. 2012 May 3;7(5):e36295. doi: 10.1371/journal.pone.0036295 (PMC3343073; doi:10.1371/journal.pone.0036295)
Supplement: Table S1 — List of strains used in this study. (PDF) [file pone.0036295.s001.pdf]

**Supplementary Table S1.** List of strains used in this study.

| Strain name | Genotype | Source |
|---|---|---|
| BY4741 | *MATa his3Δ0 leu2Δ0 met15Δ0 ura3Δ0* | Open Biosystems |
| YAG1406 | *MATa BY4741 hel1::Kan* | Open Biosystems |
| YAG1405 | *MATa BY4741 hel2::Kan* | Open Biosystems |
| YRS202 | *MATa BY4741 pep5::Kan* | Open Biosystems |
| YRS207 | *MATa BY4741 snt2::Kan* | Open Biosystems |
| YRS234 | *MATa BY4741 tom1::Kan* | Open Biosystems |
| YRS184 | *MATa BY4741 hel1::HEL1*-TAP-*HIS3* | Open Biosystems |
| YRS185 | *MATa BY4741 hel2::HEL2*-TAP-*HIS3* | Open Biosystems |
| YRS181 | *MATa BY4741 pep5::PEP5*-TAP-*HIS3* | Open Biosystems |
| YRS357 | *MATa BY4741 snt2::SNT2*-TAP-*HIS3* | Open Biosystems |
| YRS216 | *MATa BY4741 rad53::HIS3 crt1::Kan* | Singh et al., 2009a |
| YRS170 | *MATa BY4741 rad53::HIS3 crt1::Kan hel1:: Kan* | This study |
| YRS173 | *MATa BY4741 rad53::HIS3 crt1::Kan hel2:: Kan* | This study |
| YRS870 | *MATa BY4741 tom1::HIS3 snt2:: Kan* | This study |
| YRS175 | *MATa tom1::TRP1 hel1:: Kan* | This study |
| YRS177 | *MATa tom1::TRP1 hel2:: Kan* | This study |
| YRS232 | *MATa hel1::Kan Mx hel2::Kan Mx* | This study |
| YMH47 | *MATa BY4741 asf1::ASF1-FLAG3-LEU2* | This study |
| YRS192 | *MATa BY4741 hel1::Kan asf1::ASF1-FLAG3-LEU2* | This study |
| YRS193 | *MATa BY4741 hel2::Kan asf1::ASF1-FLAG3-LEU2* | This study |
| YRS189 | *MATa BY4741 pep5::Kan asf1::ASF1-FLAG3-LEU2* | This study |
| YRS335 | *MATa BY4741 snt2::Kan asf1::ASF1-FLAG3-LEU2* | This study |
| YRS337 | *MATa BY4741 rad53::HIS3 crt1::Kan asf1::ASF1-FLAG3-LEU2* | This study |
| YRS789 | *MATa BY4741 UBC4-MYC13-HIS3* | This study |
| YRS817 | *MATa BY4741 UBC4-MYC13-HIS3 +pBG1805HA-HEL1-URA3* | This study |
| YRS818 | *MATa BY4741 UBC4-MYC13-HIS3 +pBG1805HA-HEL2-URA3* | This study |
| YRS819 | *MATa BY4741 UBC4-MYC13-HIS3 +pBG1805HA-PEP5-URA3* | This study |
| YRS820 | *MATa BY4741 UBC4-MYC13-HIS3 +pBG1805HA-SNT2-URA3* | This study |
| YRS792 | *MATa BY4741 HEL1-MYC13-HIS3 +pRS416-RAD53-FLAG* | This study |
| YRS793 | *MATa BY4741 HEL2-MYC13-HIS3 + pRS416-RAD53-FLAG* | This study |
| YRS823 | *MATa BY4741 pep5::PEP5*-TAP-*HIS3 + pRS416-RAD53-FLAG* | This study |
| YRS824 | *MATa BY4741 snt2::SNT2*-TAP-*HIS3 + pRS416-RAD53-FLAG* | This study |
| YRS794 | *MATa BY4741 hel1::Kan ubc4::UBC4-MYC13-HIS3* | This study |
| YRS795 | *MATa BY4741 hel2::Kan ubc4::UBC4-MYC13-HIS3* | This study |
| YRS796 | *MATa BY4741 pep5::Kan ubc4::UBC4-MYC13-HIS3* | This study |
| YRS797 | *MATa BY4741 snt2::Kan ubc4::UBC4-MYC13-HIS3* | This study |
| YRS79 | *MATa BY4741 tom1::Kan ubc4::UBC4-MYC13-HIS3* | This study |
| YRS99 | *MATa BY4741* +pYES2-HTH | This study |
| YRS100 | *MATa BY4741* +pYES2-HTH-*HHT2* | This study |
| YRS324 | *MATa BY4741 hel1::Kan* +pYES2-HTH | This study |
| YRS325 | *MATa BY4741 hel1::Kan* +pYES2-HTH-*HHT2* | This study |
| YNG15 | *MATa BY4741 hel2::Kan* +pYES2-HTH | This study |
| YNG16 | *MATa BY4741 hel2::Kan* +pYES2-HTH-*HHT2* | This study |
| YRS314 | *MATa BY4741 snt2::Kan* +pYES2-HTH | This study |
| YRS315 | *MATa BY4741 snt2::Kan* +pYES2-HTH-*HHT2* | This study |

| | | |
|---|---|---|
| YRS125 | *MATa BY4741 pep5::Kan* +pYES2-HTH | This study |
| YRS126 | *MATa BY4741 pep5::Kan* +pYES2-HTH-*HHT2* | This study |
| YJJ75 | *MATa BY4741 tom1::Kan* +pYES2-HTH | This study |
| YJJ76 | *MATa BY4741 tom1::Kan* +pYES2-HTH-*HHT2* | This study |
| YRS338 | *MATa BY4741 rad53::HIS3 crt1::Kan* +pYES2-HTH | This study |
| YRS339 | *MATa BY4741 rad53::HIS3 crt1::Kan* +pYES2-HTH-*HHT2* | This study |
| YMG3 | *MATa BY4741 hel1::Kan +pBG1805-HA- hel*1-$r_1$-*URA3* | This study |
| YMG4 | *MATa BY4741 hel1::Kan +pBG1805-HA- hel*1-$r_2$-*URA3* | This study |
| YMG5 | *MATa BY4741 hel1::Kan +pBG1805-HA- hel*1-$r_1r_2$-*URA3* | This study |
| YMG6 | *MATa BY4741 hel2::Kan +pBG1805-HA- hel*2-r-*URA3* | This study |
| YMG7 | *MATa BY4741 snt2::Kan +pBG1805-HA- snt*2-$r_2$-*URA3* | This study |
| YRS696 | *MATa BY4741 hel1::Kan* + pYES6/CT -HA-*HHT2* + *pBG1805-HA-hel*1-$r_1$-*URA3* | This study |
| YRS698 | *MATa BY4741 hel1::Kan* + pYES6/CT -HA-*HHT2* + *pBG1805-HA-hel*1-$r_2$-*URA3* | This study |
| YRS700 | *MATa BY4741 hel1::Kan* + pYES6/CT -HA-*HHT2* + *pBG1805-HA-hel*1-$r_1r_2$-*URA3* | This study |
| YRS701 | *MATa BY4741 hel2::Kan* + pYES6/CT -HA-*HHT2* + *pBG1805-HA-hel*2-r-*URA3* | This study |
| YRS767 | *MATa BY4741 snt2::Kan* + pYES6/CT -HA-*HHT2* + *pBG1805-HA-snt*2-$r_2$-*URA3* | This study |
| YRS703 | *MATa BY4741* + pYES6/CT -HA-*HHT2* | This study |
